# Pharmacokinetics/pharmacodynamics of KSP-1007 in combination with meropenem against carbapenemase-producing gram-negative bacteria in a neutropenic murine thigh infection model

**DOI:** 10.1128/aac.01852-25

**Published:** 2026-03-03

**Authors:** Koji Takemoto, Mami Kamada, Keizo Matsushita, Hidehito Matsui, Jun Hidaka, Hideaki Hanaki

**Affiliations:** 1 Corporate Regulatory Compliance & Quality Assurance Division, Sumitomo Pharma Co., Ltd. 38293 , Osaka, Japan; 2 Research and Development Division, Sumitomo Pharma Co., Ltd. 38293 , Osaka, Japan; 3 Ōmura Satoshi Memorial Institute, Kitasato University, Tokyo, Japan; Providence Portland Medical Center, Portland, Oregon, USA

**Keywords:** antibiotic resistance, carbapenems, beta-lactamases

## Abstract

KSP-1007 is a novel bicyclic boronate-based broad-spectrum β-lactamase inhibitor that improves meropenem (MEM) efficacy against gram-negative bacteria (GNB) producing serine- and metallo-type carbapenemases. We investigated which pharmacokinetics (PK)/pharmacodynamics (PD) parameters of KSP-1007 correlated with MEM/KSP-1007 efficacy against carbapenemase-producing (CP) *Enterobacterales* (CPE) and *Acinetobacter baumannii* (CPAB) in a neutropenic murine thigh infection model. Infected neutropenic mice were subcutaneously treated with MEM every 3 h for 24 h alone or in combination with KSP-1007 administered at 3-, 6-, 12-, or 24-h dosing intervals. The bacterial burden in the thigh was assessed 2 h and 26 h after infection. The percentage of the dosing interval at which the free drug concentration exceeded the threshold concentration (%*f*T>C_T_) was a good predictor of activity against serine- and metallo-CPE and CPAB based on coefficients of determinations (R^2^ = 0.5–0.8) and a visual inspection of the plots showing %*f*T>C_T_ against the change in log_10_ CFU/thigh, although the area under the 24-h free drug concentration time-curve yielded similar R^2^ values. The required values for %*f*T>C_T_ at 0.25–4 μg/mL were 20.4%–4.2% (stasis) and 45.1%–14.1% (1-log_10_ kill) for serine-CPE and 48.6%–13.3% (stasis) and 97.9%–42.3% (1-log_10_ kill) for metallo-CPE. The corresponding values for CPAB at thresholds of 0.5–8 μg/mL were 15.3%–4.3% and 33.9%–8.7%. Frequent dosing of KSP 1007 maximized its potentiation of MEM activity against most of the tested CP-GNB strains. Therefore, the *in vivo* efficacy of KSP-1007 with MEM against CP-GNB was not dependent on the peak free drug concentration of KSP-1007, making KSP-1007 an optimal partner for MEM, which exhibits time-dependent activity.

## INTRODUCTION

One strategy to treat carbapenemase-producing (CP) *Enterobacterales* (CPE) and *Acinetobacter baumannii* (CPAB) infections, which are major global health concerns, is to combine a β-lactams with a β-lactamase inhibitor (1, 2). KSP-1007 is a novel bicyclic boronate-based broad-spectrum β-lactamase inhibitor that is being developed in combination with meropenem (MEM) for the treatment of infections caused by carbapenem-resistant gram-negative bacteria (GNB) (3). KSP-1007 inhibits the following β-lactamases: class A (*Klebsiella pneumoniae* carbapenemases [KPCs], Cefotaximase-Munichs [CTX-Ms], sulfhydryl variables [SHVs], and Temoniera-10 [TEM-10]); class B (New Delhi metallo-β-lactamases [NDMs], Verona integron-encoded metallo-β-lactamases [VIMs], and imipenemase-1 [IMP-1]); class C (AmpC-type-17 [ACT-17] and *Pseudomonas*-derived cephalosporinase-11 [PDC-11]); and class D (oxacillinases [OXAs]) and exhibits good activity against CP-GNB in combination with MEM (3).

A pharmacokinetics (PK)/pharmacodynamics (PD) analysis of antimicrobial agents is important for designing appropriate clinical dosages and administration (4). The pharmacological characteristics of antimicrobial agents have been classified into the following types of PK/PD indices: the peak free drug concentration (*f*C_max_)/MIC ratio (*f*C_max_/MIC), the area under the 24-h free drug concentration time-curve (*f*AUC)/MIC ratio (*f*AUC/MIC), and the ratio of the time period in which the free drug concentration surpasses the MIC in the dosing interval (%*f*T>MIC) (4). Using animal infection models, it is important to establish which PK/PD parameters correlate with antimicrobial activity and the magnitude of the index necessary to achieve bacteriostatic and bactericidal efficacies (4). β-Lactam antibiotics including MEM exhibit time-dependent antibacterial activity, and their PK/PD index is %*f*T>MIC (4). In other words, prolonging the infusion time and shortening the dosing interval increase the efficacy of β-lactams (5). Among β-lactamase inhibitors used in combination with β-lactams, the %*f*T>threshold concentration (%*f*T>C_T_) for tazobactam and avibactam and the *f*AUC/MIC for relebactam and vaborbactam have been identified as the PK/PD index (6–8). However, since the PK/PD index of KSP-1007 is still unknown, we herein investigated which PK/PD parameters of KSP-1007 correlated with the efficacy of KSP-1007 against CP-GNB in combination with MEM in a neutropenic murine thigh infection model.

## RESULTS

### *In vivo* efficacy against CPE and CPAB in a neutropenic murine thigh infection model

The MICs and β-lactamases harbored by GNB strains, along with the dosing regimens of MEM and KSP-1007 used in a murine thigh infection model, are summarized in Table 1. In all 14 models treated with MEM alone, bacterial counts in the thigh were higher by 0.56 to 2.95 log_10_ than at treatment initiation (see Table S1 in the supplemental material). When the total daily dose of KSP-1007 was held constant, the effective dose corresponding to 50% of *E*_max_ − *E*_min_ (ED_50_) values of every 3 h (q3h) regimen were lower than those of less-frequent regimens for most strains (7/9 CPE, except *K. pneumoniae* ATCC BAA-1902 and ATCC BAA-2473; 5/5 CPAB) (see Table S1 in the supplemental material), suggesting that maintaining the concentration of KSP-1007 above a certain threshold contributed to potentiating the activity of MEM. KSP-1007 enhanced the *in vivo* antimicrobial activity of MEM in a dose-dependent manner in this infection model (Jonckheere–Terpstra test, *P* < 0.05) for the q3h, every 6 h (q6h), and every 12 h (q12h) regimens, except for q6h for *K. pneumoniae* ATCC BAA-1902 and q12h for *K. pneumoniae* CDC-40 and CDC-138, as exemplified by the representative results shown in Fig. S1 to S4 in the supplemental material.

**TABLE 1:** Summary of bacterial strains and dosing regimens of MEM and KSP-1007 used in a murine thigh infection model

Strain	Carbapenemase	MICs of MEM (μg/mL) with KSP-1007 (μg/mL) at				MEM dose (mg/kg) q3h	KSP-1007	
		Alone	4	8	16		Total doses (mg/kg/day)	Intervals (h)
*K. pneumoniae* ATCC BAA-2344	KPC-2	4	0.015	0.015	0.015	5	16, 4, and 1	24, 12, 6, and 3
*K. pneumoniae* ATCC BAA-1902	KPC-3	64	0.12	0.12	0.06	5	960^*a*^, 240, and 60	24, 12, 6, and 3
*K. pneumoniae* CDC-113	KPC-3	256	16	0.12	0.12	100	480, 240, 120, and 60	12, 6, and 3
*K. pneumoniae* KUB3606	KPC-38	256	32	0.5	0.25	100	960, 240, and 60	12, 6, and 3
*K. pneumoniae* KUB3166	IMP-1	0.5	0.03	0.03	0.015	5	240, 60, and 15	24, 12, 6, and 3
*K. pneumoniae* ATCC BAA-2473	NDM-1	64	16	4	0.25	5	960^*a*^, 240, and 60	24, 12, 6, and 3
*K. pneumoniae* CDC-40	VIM-27	64	16	8	1	50	480, 240, 120, and 60	12, 6, and 3
*K. pneumoniae* CDC-68	NDM-1 and OXA-232	128	32	1	0.25	100	480, 120, 60, and 15	12, 6, and 3
*K. pneumoniae* CDC-138	NDM-7	256	128	32	1	100	480, 240, 120, and 60	12, 6, and 3
*A*. *baumannii* CDC-277	OXA-24 and OXA-65	128	8	2	2	100	960^*a*^, 240, and 60	24, 12, 6, and 3
*A*. *baumannii* CDC-83	NDM-1, OXA-23, and OXA-69	128	16	2	1	100	960^*a*^, 240, and 60	24, 12, 6, and 3
*A*. *baumannii* CDC-289	OXA-66 and OXA-72	128	32	8	4	100	480, 240, and 60	24, 12, 6, and 3
*A*. *baumannii* CDC-301	OXA-66 and OXA-72	>128	64	8	2	100	960^*a*^, 240, and 60	24, 12, 6, and 3
*A*. *baumannii* CDC-293	OXA-66 and OXA-72	>128	128	16	1	100	960^*a*^, 240, and 60	24, 12, 6, and 3

^*a*^ 960 mg/kg q24h was not performed.

### *In vivo* pharmacodynamic target assessment of KSP-1007 against CPE in a murine thigh infection model

**Fig 1:**
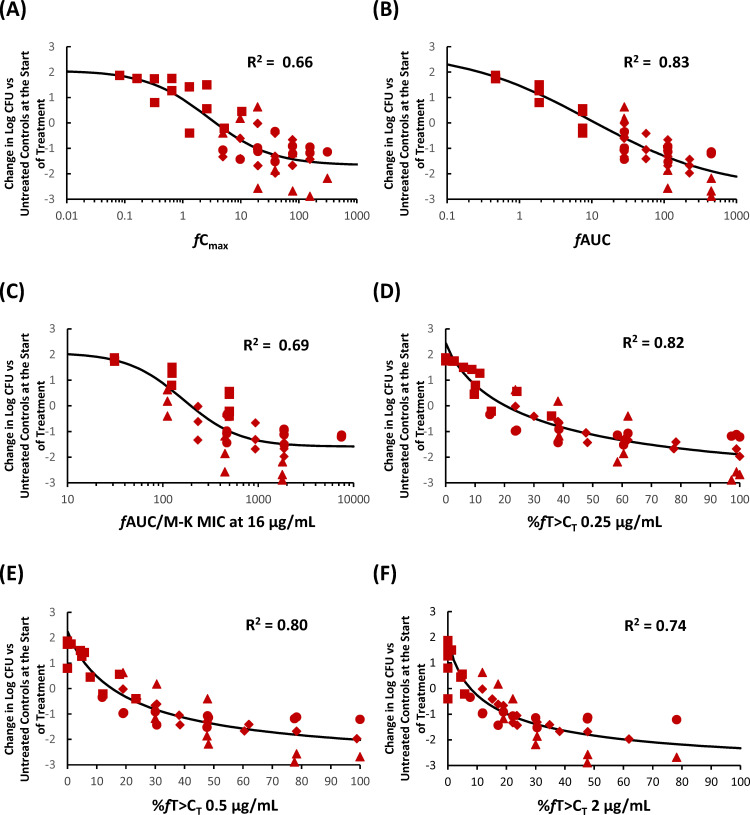
Representative PK/PD relationship for KSP-1007 in combination with MEM in a murine thigh infection model infected with *K. pneumoniae* (4 strains of serine-CPE). Relationship between *f*C_max_ (**A**), *f*AUC (**B**), %*f*AUC/M-K MIC at 16 μg/mL (**C**), %*f*T>C_T_ 0.25 μg/mL (**D**), %*f*T>C_T_ 0.5 μg/mL (**E**), or %*f*T>C_T_ 2 μg/mL (**F**) and the change in log_10_ CFU/thigh relative to treatment initiation. Red squares, red circles, red diamonds, and red triangles represent *K. pneumoniae* ATCC BAA-2344, ATCC BAA-1902, CDC-113, and KUB3606, respectively.

**Fig 2:**
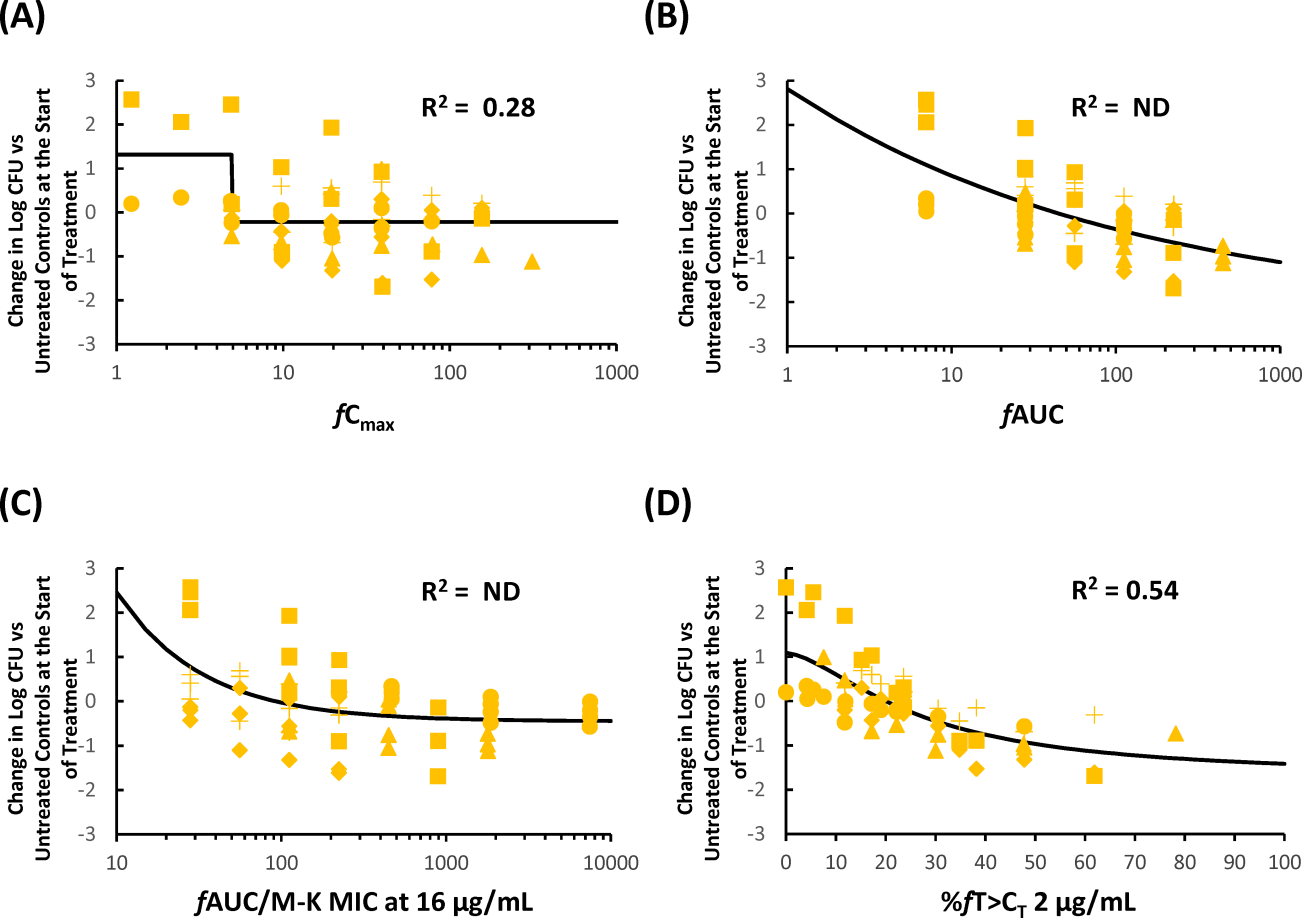
Representative PK/PD relationship for KSP-1007 in combination with MEM in a murine thigh infection model infected with *K. pneumoniae* (5 strains of metallo-CPE). Relationship between *f*C_max_ (**A**), *f*AUC (**B**), %*f*AUC/M-K MIC at 16 μg/mL (**C**), or %*f*T>C_T_ 2 μg/mL (**D**) and the change in log_10_ CFU/thigh relative to treatment initiation. Yellow circles, yellow triangles, yellow crosses, yellow squares, and yellow diamonds represent *K. pneumoniae* KUB3166, ATCC BAA-2473, CDC-40, CDC-68, and CDC-138, respectively. ND, not determined.

**Fig 3:**
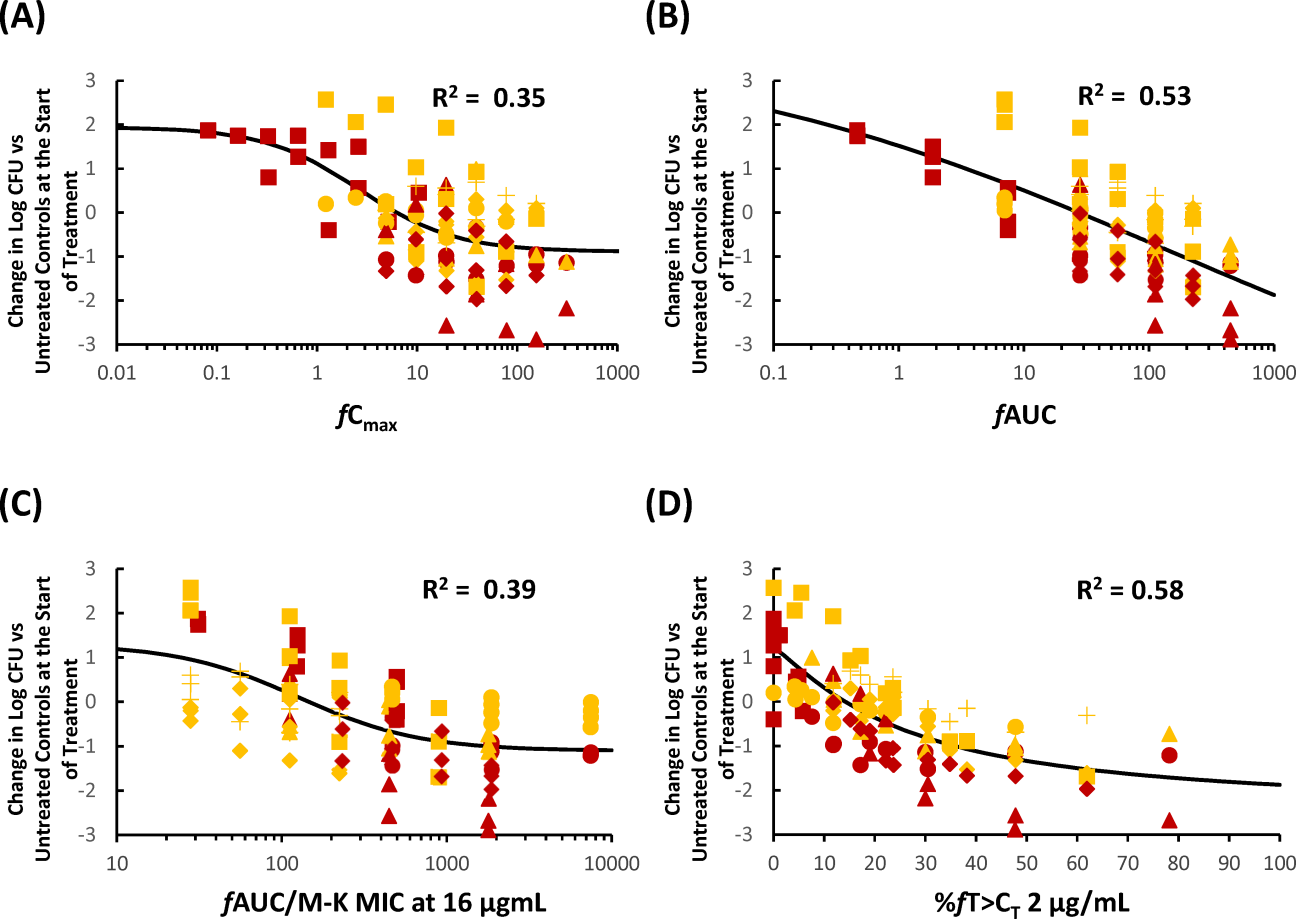
Representative PK/PD relationship for KSP-1007 in combination with MEM in a murine thigh infection model infected with *K. pneumoniae* (9 strains of CPE). Relationship between *f*C_max_ (**A**), *f*AUC (**B**), %*f*AUC/M-K MIC at 16 μg/mL (**C**), or %*f*T>C_T_ 2 μg/mL (**D**) and the change in log_10_ CFU/thigh relative to treatment initiation. Red squares, red circles, red diamonds, red triangles, yellow circles, yellow triangles, yellow crosses, yellow squares, and yellow diamonds represent *K. pneumoniae* ATCC BAA-2344, ATCC BAA-1902, CDC-113, KUB3606, KUB3166, ATCC BAA-2473, CDC-40, CDC-68, and CDC-138, respectively.

The coefficients of determinations (R^2^) between the efficacy of KSP-1007 in combination with MEM against CPE and each PK/PD parameter of KSP-1007 are listed in Table 2. The relationships between changes in log_10_ CFU/thigh and a representative PK/PD parameter are shown in Fig. 1 to 3. In the 4 strains of serine-CPE, %*f*T>C_T_ and *f*AUC were good predictors of the *in vivo* antimicrobial activity of MEM/KSP-1007 based on R^2^ values (0.70–0.82 for %*f*T>C_T_ at 0.25–4 μg/mL and 0.83 for *f*AUC) and a visual inspection of the plots showing %*f*T>C_T_ or *f*AUC against changes in log_10_ CFU/thigh (Fig. 1). In the 5 strains of metallo-CPE, %*f*T>C_T_ was the best predictor (R^2^ around 0.5 at tested C_T_). Since the serine- and metallo-CPE subsets both correlated well with %*f*T>C_T_, a pooled analysis of all 9 strains of CPE confirmed that %*f*T>C_T_ remained a good overall predictor (R^2^ = 0.54–0.58).

**TABLE 2:** PK/PD parameters and coefficients of determinations

PK/PD parameters of KSP-1007	Coefficients of determinations			
	Four strains of serine-CPE	Five strains of metallo-CPE	Nine strains of CPE	Five strains of CPAB
*f*AUC	0.83	ND^*a*^	0.53	0.63
*f*AUC/M-K MIC at 4 μg/mL	0.00	0.18	0.10	0.48
*f*AUC/M-K MIC at 8 μg/mL	0.56	0.00	0.23	0.50
*f*AUC/M-K MIC at 16 μg/mL	0.69	ND^*a*^	0.39	0.43
*f*C_max_	0.66	0.28	0.35	0.21
*f*C_max_/M-K MIC at 4 μg/mL	−0.00	0.08	0.05	0.20
*f*C_max_/M-K MIC at 8 μg/mL	0.28	ND^*a*^	0.10	ND^*a*^
*f*C_max_/M-K MIC at 16 μg/mL	0.39	0.07	0.18	ND^*a*^
%*f*T>C_T_ 0.25 μg/mL	0.82	0.48	0.55	NT^*b*^
%*f*T>C_T_ 0.5 μg/mL	0.80	0.50	0.55	0.54
%*f*T>C_T_ 1 μg/mL	0.70	0.52	0.57	0.55
%*f*T>C_T_ 2 μg/mL	0.74	0.54	0.58	0.58
%*f*T>C_T_ 4 μg/mL	0.80	0.51	0.54	0.61
%*f*T>C_T_ 8 μg/mL	NT^*b*^	NT^*b*^	NT^*b*^	0.54
%*f*T>M K MIC at 4 μg/mL	−0.16	−0.05	−0.93	0.11
%*f*T>M K MIC at 8 μg/mL	0.52	0.01	0.27	0.23
%*f*T>M K MIC at 16 μg/mL	0.61	0.39	0.49	0.46

^*a*^ ND, not determined.

^*b*^ NT, not tested.

The magnitude of %*f*T>C_T_ and *f*AUC required for a bacteriostatic effect and 1-log_10_ of bacterial killing are summarized in Table 3. The required values of %*f*T>C_T_ at thresholds of 0.25–4 μg/mL were 20.4%–4.2% (stasis) and 45.1%–14.1% (1-log_10_ kill) for serine-CPE, 48.6%–13.3% and 97.9%–42.3% for metallo-CPE, and 33.3%–8.9% and 75.1%–27.0% for pooled CPE. Corresponding *f*AUC targets were 11.3 and 56.6 μg·h/mL for serine-CPE, 44.9 and 685 μg·h/mL for metallo-CPE, and 27.5 and 186 μg·h/mL for pooled CPE (Table 3).

**TABLE 3:** PK/PD parameters and corresponding magnitudes required for effects

Organism (n)	%*f*T>C_T_, μg/mL						*f*AUC (μg·h/mL)
	0.25	0.5	1	2	4	8	
Stasis							
Serine-CPE (4)	20.4	15.2	10.5	7.7	4.2	NT^*a*^	11.3
Metallo-CPE (5)	48.6	37.1	27.2	20.0	13.3	NT^*a*^	44.9
CPE (9)	33.3	25.6	19.3	14.1	8.9	NT^*a*^	27.5
CPAB (5)	NT^*a*^	15.3	13.0	11.0	7.2	4.3	31.7
1-log kill							
Serine-CPE (4)	45.1	35.0	26.5	20.4	14.1	NT^*a*^	56.6
Metallo-CPE (5)	97.9	82.7	67.4	51.9	42.3	NT^*a*^	685
CPE (9)	75.1	60.8	46.9	35.8	27.0	NT^*a*^	186
CPAB (5)	NT^*a*^	33.9	26.3	20.1	13.3	8.7	73.6

^*a*^ NT, not tested.

### *In vivo* pharmacodynamic target assessment of KSP-1007 against CPAB in a murine thigh infection model

**Fig 4:**
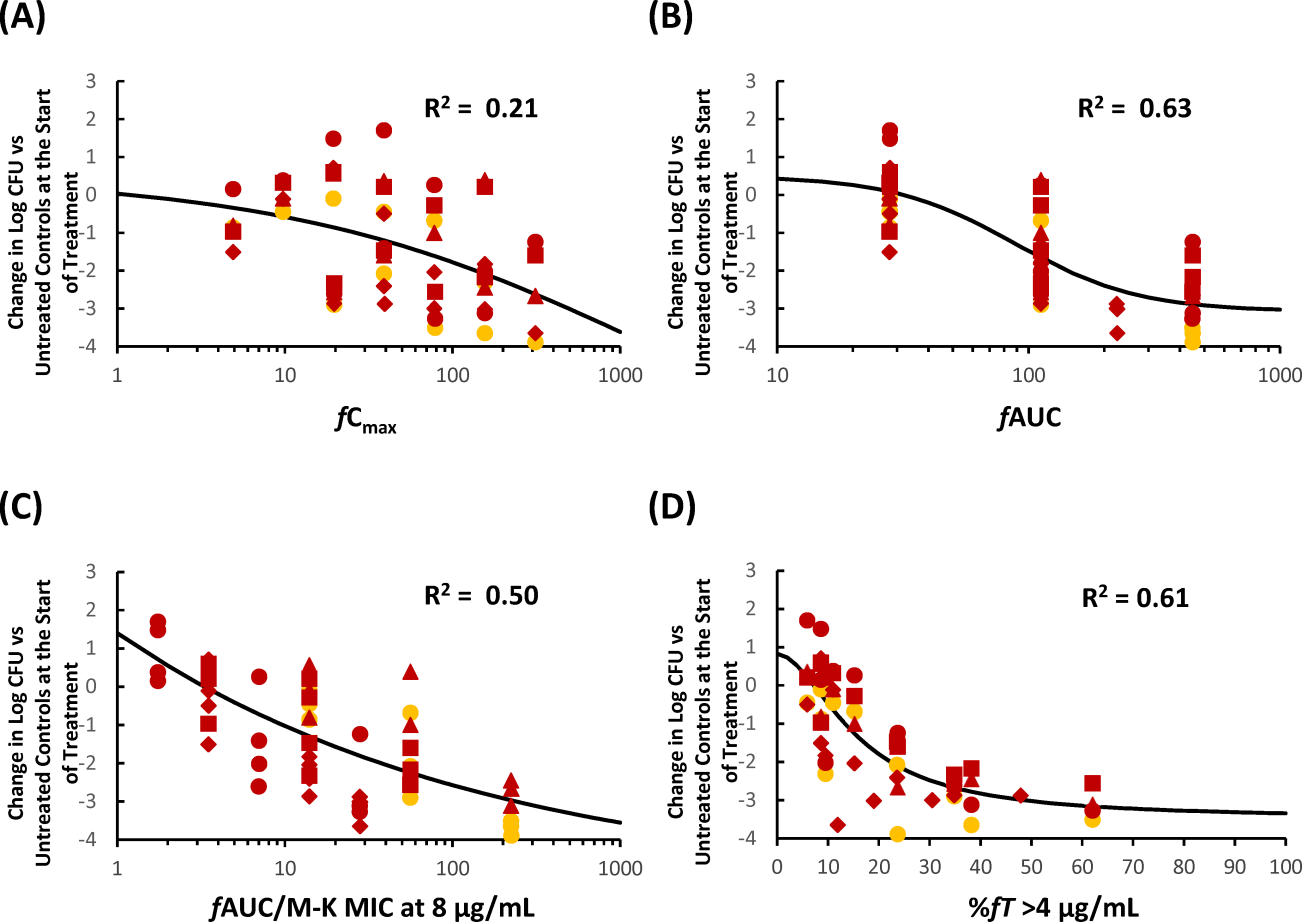
Representative PK/PD relationship for KSP-1007 in combination with MEM in a murine thigh infection model infected with *A. baumannii*. Relationship between *f*C_max_ (**A**), *f*AUC (**B**), %*f*AUC/M-K MIC at 8 μg/mL (**C**), or %*f*T>C_T_ 4 μg/mL (**D**) and the change in log_10_ CFU/thigh relative to treatment initiation. Red squares, red circles, red triangles, red diamonds, and yellow circles represent *A. baumannii* CDC-301, CDC-293, CDC-277, CDC-289, and CDC-83, respectively.

Table 2 summarizes R^2^ values reflecting the correlations between antimicrobial efficacy and each PK/PD parameter. The parameter %*f*T>C_T_ at 0.5–8 μg/mL was also identified as a key factor affecting antimicrobial activity, supported by R^2^ values of 0.54–0.61 and a visual inspection of the relationship between %*f*T>C_T_ and the change in log_10_ CFU/thigh; however, *f*AUC yielded a slightly higher R^2^ value of 0.63 (Fig. 4). The %*f*T>C_T_ at 0.5–8 μg/mL values required to achieve bacteriostatic and bactericidal effects were estimated to be 15.3%–4.3% and 33.9%–8.7%, respectively (Table 3). Corresponding *f*AUC targets were 31.7 and 73.6 μg·h/mL.

## DISCUSSION

To enhance the clinical efficacy of MEM, dose escalations and prolonged infusions have both been adopted (5). For example, under the dosing regimen of 2 g every 8 h (q8h) as a 3-h infusion, the PK/PD breakpoint MIC for MEM is considered to be 8 μg/mL (9), which is higher than the 2 μg/mL breakpoint observed with the standard 1 g q8h as a 30-min infusion (9, 10). In the present study, we investigated the PK/PD relationship of KSP-1007 in combination with MEM for its efficacy against CP-GNPs using a neutropenic murine thigh infection model. When the total daily dose of KSP-1007 was fixed, more frequent dosing resulted in higher bactericidal activity against most strains than less frequent dosing. This result suggests that KSP-1007 provides a time-dependent enhancement in MEM activity rather than a C_max_-dependent enhancement. This pharmacodynamic profile aligns well with that of MEM, supporting the rationale for combining KSP-1007 with MEM. Based on these findings, a phase 1 clinical trial on MEM (2 g q8h as a 3-h infusion) in combination with KSP-1007 was conducted (11).

We administered 100 mg/kg of cilastatin, a dehydropeptidase-I inhibitor, with each administration of MEM to prevent MEM degradation by this rodent enzyme (12, 13) in our murine thigh infection model. The *f*C_max_ and *f*AUC_0–24 h_ of MEM at 100 mg/kg with cilastatin 100 mg/kg were 85.4 µg/mL and 424 µg·h/mL, respectively (see Table S3 in the supplemental material). Previous reports have indicated that MEM exhibits 2% protein binding in humans (14) and also that the administration of 2 g q8h as a 3-h infusion results in a C_max_ of 46.0 µg/mL (15) and *f*AUC_0–24 h_ of 402 µg·h/mL (14), suggesting that the free plasma PK of MEM in this human dose regimen is approximately equivalent to those achieved with 100 mg/kg q3h in mice over a 24-h period. In mice, MEM administered at 100 mg/kg q3h achieved >40% of %*f*T>8 µg/mL (see Table S3 in the supplemental material), which is similar to the 56% of %*f*T>8 µg/mL observed in humans receiving 2 g q8h as a 3-h infusion (14). These results support the use of 100 mg/kg q3h in mice as a pharmacokinetically equivalent model to 6 g/day in humans. Without the coadministration of cilastatin, ≥40% of %*f*T>8 µg/mL is achieved with MEM 300 mg/kg every 2 h (14). The C_max_ and *f*AUC_0–24 h_ in this dosage regimen were 245 µg/mL and 1,572 µg·h/mL, respectively, which were approximately 5- and 4-fold higher, respectively, than those for 6 g/day in humans (14). Therefore, from the viewpoint of mimicking the MEM plasma PK in humans, the cilastatin combination is useful.

In the murine thigh infection model, we used highly resistant isolates of *K. pneumoniae* (CDC-113 and CDC-138) and *A. baumannii* (CDC-301 and CDC-83), which exhibited MEM MICs of ≥128 µg/mL (see Table 1; Fig. S1 to S4 in the supplemental material). The addition of 8 μg/mL KSP-1007 reduced MEM MIC to 0.12–32 μg/mL, while the addition of 16 μg/mL KSP-1007 restored MEM MIC to a clinically susceptible level (0.12 to 2 μg/mL). The treatment with MEM at 100 mg/mL q3h over 24 h, which mimics exposure to 2 g q8h in humans, resulted in %*f*T>MIC (≥128 µg/mL) of 0% (see Table S3 in the supplemental material) and approximately 1-log_10_ bacterial growth from the baseline. The co-administration of KSP-1007 at 30 mg/kg q3h resulted in *f*AUC_0–24 h_ of 112 μg·h/mL, which was similar to the exposure predicted in humans following a q8h dose of 0.5 g (11), and achieved %*f*T>C_T_ 2 μg/mL of 47.8% and %*f*T>C_T_ 4 μg/mL of 34.8% (see Table S2 in the supplemental material). This combination increased the %*f*T>MIC of MEM and resulted in a bactericidal effect. Based on pharmacokinetic data from the phase 1 clinical trial, the predicted %*f*T>C_T_ values for KSP-1007 at 0.5 g q8h are >90% at 2 μg/mL and >50% at 4 μg/mL (unpublished in-house data). Therefore, the combination of MEM 2 g and KSP-1007 0.5 g q8h as a 3-h infusion is expected to be effective against such highly resistant strains.

Bicyclic boronate-based β-lactamase inhibitors exhibit distinct inhibitory mechanisms: they form a reversible covalent bond with serine β-lactamases, whereas they act as competitive inhibitors against metallo-β-lactamases (16, 17). The inhibitory activity of KSP-1007 is stronger against the former, and the MICs of MEM in combination with KSP-1007 are lower against serine-CPE than against metallo-CPE (3). These *in vitro* differences are likely reflected in the distinctions in PK/PD target values required to achieve stasis and 1-log kill between serine-CPE and metallo-CPE observed in the present study. Of note, the R^2^ values for PK/PD parameters associated with the efficacy of KSP-1007 under MEM against metallo-CPE were lower than those for serine-CPE, which indicates that further investigations with an expanded panel of metallo-CPE strains will be important for obtaining a more detailed understanding of these relationships.

Given that the pharmacological profile of MEM/KSP-1007 (3) suggests its potential as a therapeutic option for hospital-acquired pneumonia caused by CP-GNB, it is important to investigate the PK/PD parameters of KSP-1007 correlating with the efficacy of KSP-1007 against CP-GNB in combination with MEM in a murine lung infection model. In a preliminary lung infection study (Fig. S5B and the supplemental methods in the supplemental material), KSP-1007 exhibited good pulmonary penetration, as indicated by the high AUC ratio of KSP-1007 in the epithelial lining fluid to that in plasma at doses of 30 to 240 mg/kg (ranged from 68 to 100%). Based on this result, the efficacy of KSP-1007 in combination with MEM in the lung model was considered to be assessable using plasma-based PK/PD parameters. In the preliminary lung infection study (Table S4 and the supplemental methods in the supplemental material), although only one strain of *A. baumannii* CDC-277 was used and MEM alone showed near stasis (0.1-log_10_ growth), the MEM/KSP-1007 (240 and 480 mg/kg/day) combination achieved an approximately 4-log_10_ reduction in the lung from baseline at treatment initiation. Furthermore, the fractionated dosing of KSP-1007 did not diminish its antibacterial effects in the lungs, suggesting that the driver of efficacy is not *f*C_max_. In the future, lung infection studies using multiple CP-GNP strains are warranted to establish whether %*f*T>C_T_ or *f*AUC is the most relevant index.

Ultimately, PK/PD analyses of MEM in combination with KSP-1007 need to be conducted using infection models that simulate human plasma exposure at clinical doses, as demonstrated by Nicolau et al. (18). An important result of the present study is that the fractionated dosing of KSP-1007 in combination with MEM maximized antibacterial efficacy against most strains, providing valuable insights into appropriate dosing strategies for future clinical trials. Among boronic acid-based β-lactamase inhibitors, such as vaborbactam and taniborbactam, *f*AUC/MIC has been shown to correlate with antibacterial activity (7, 18). In the case of xeruborbactam, another boronic acid compound, %*f*T>C_T_ at 1 μg/mL and *f*AUC have both been reported to correlate with efficacy (19). The PK/PD profile of KSP-1007 appears to be similar to that of xeruborbactam and is consistent with other boronic acid compounds, in that its activity is not dependent on *f*C_max_.

In conclusion, the present study established %*f*T>C_T_ as a key PK/PD index for KSP-1007 efficacy and demonstrated that frequent dosing maximized its potentiation of MEM activity against most CP-GNB strains. These results provide a pharmacological foundation for designing effective clinical regimens against CP-GNB infections.

## MATERIALS AND METHODS

### Organisms

Four strains of CP-*K. pneumoniae* and five strains of CP-*A. baumannii* were obtained from the CDC & FDA Antibiotic Resistance Isolate Bank via the National Institute of Infectious Diseases, Japan. Three strains of CP-*K. pneumoniae* were obtained from the American Type Culture Collection (ATCC). Two strains of CP-*K. pneumoniae* were clinical isolates collected in Japan.

### Antibacterial agent and β-lactamase inhibitor

KSP-1007 and MEM hydrate were synthesized at Sumitomo Pharma Co., Ltd.

### MIC testing

MICs were assessed by the broth microdilution method according to CLSI (20). Three fixed concentrations of KSP-1007 (4, 8, and 16 µg/mL) were evaluated in combination with MEM (concentration range 0.004 to 512 µg/mL for *K. pneumoniae* and 0.25 to 128 µg/mL for *A. baumannii*).

### Murine thigh infection models

Four-week-old male Slc:ICR mice were purchased from Japan SLC, Inc. (Shizuoka, Japan) and were used at 5 weeks of age. The organisms were grown at 35°C overnight on Mueller Hinton agar (MHA). Cells were then suspended in sterile saline and adjusted to 0.5 McFarland. An overnight culture was prepared by inoculating 4 mL cation-adjusted Mueller Hinton broth (CAMHB) with 40 μL of the bacterial suspension at 36°C with agitation at 40 rpm. The overnight culture was diluted with fresh CAMHB, adjusted to 1.5 McFarland, and incubated at 36°C for 2 h with agitation at 40 rpm. The bacterial culture was diluted in sterile saline to achieve a concentration of approximately 1 × 10^7^ CFU/mL. To induce neutropenia, mice were intraperitoneally administered 150 and 100 mg/kg cyclophosphamide (Shionogi and Co., Ltd., Osaka, Japan) 4 days and 1 day before infection, respectively. As previously described (21), cyclophosphamide-induced neutropenic mice were inoculated with 0.1 mL of the bacterial suspensions into the left gastrocnemius muscles (approximately 1 × 10^6^ CFU/mouse) 2 h before therapy. Infected mice were subcutaneously treated with MEM from 5 to 100 mg/kg q3h for 24 h with and without several dosing regimens of KSP-1007 administered at 3-, 6-, 12-, or 24-h dosing intervals (total doses 1 to 960 mg/kg/day). At each administration, 100 mg/kg of cilastatin (BLD Pharmatech Ltd., Shanghai, China), a dehydropeptidase-I inhibitor, was co-administered to prevent MEM degradation by this rodent enzyme (12, 13). The control group was subcutaneously administered sterile saline q3h for 24 h. The bacterial burden in the thighs was assessed 2 h and 26 h after infection. Mice were sacrificed by bleeding from the heart under isoflurane anesthesia. The thigh was excised and homogenized in 1 mL of sterile saline with a Multi-Beads shocker (Yasui Kikai Co., Osaka, Japan). Approximately 10-fold serial dilutions of muscle homogenates were prepared using sterile saline, with 0.1 mL of each dilution being plated onto MHA. The plates were then incubated at 35°C for 1 day, and colonies were then enumerated. The mean change in log_10_ CFU/thigh at 24 h relative to treatment initiation for 4 mice per group was used as efficacy data.

To investigate the PK/PD parameters of KSP-1007, the dose regimens of MEM that were not effective against the target pathogens when administered alone were selected regardless of the clinical dose regimen of MEM. That is, we considered 100 mg/kg q3h to approximate human MEM 2 g q8h administered as a 3-h infusion in terms of *f*C_max_, *f*AUC_0–24h_, and %*f*T>8 µg/mL (see Discussion). Based on this, in preliminary experiments using the neutropenic murine thigh infection model, dose explorations up to 100 mg/kg q3h were conducted and, for each strain, the highest MEM dose that met the ineffective criterion (i.e., ≥0.5 log_10_ CFU/thigh increase over 24 h relative to the baseline at 2 h post-infection) was then identified. In the present study, MEM at 50 to 100 mg/kg q3h was used in the 10 models, and MEM 5 mg/kg q3h was used in the remaining 4 models.

### Measurement of MEM and KSP-1007 concentrations in plasma

A single dose of MEM (5, 20, or 100 mg/kg) was administered subcutaneously 2 h after infection in the thigh infection model, with cilastatin (100 mg/kg) being co-administered. A single dose of KSP-1007 was administered subcutaneously 2 h after infection: 5, 20, or 100 mg/kg in the thigh infection model. Blood samples were collected at 8 time points post-dosing via cardiac puncture under anesthesia and placed in a heparinized tube (MEM: 0.083, 0.25, 0.5, 1, 1.5, 2, 3, and 4 h; KSP-1007: 0.083, 0.25, 0.5, 1, 2, 4, 8, and 24 h). Plasma was separated by centrifugation and stored at −80°C until analyzed. The plasma concentrations of MEM and KSP-1007 were measured by liquid chromatography-tandem mass spectrometry.

The pharmacokinetic parameters of MEM were estimated using a one-compartment model, and those of KSP-1007 were estimated using a two-compartment model. All analyses were performed using Phoenix WinNonlin version 7.0 (Certara L.P., Radnor, PA).

### PK/PD relationship and magnitude of PK/PD parameters

To calculate unbound concentrations in mouse plasma, a protein binding rate of 18.6% assessed by ultrafiltration was used for KSP-1007, while a reported value of 10% was used for MEM (22). The following PK/PD parameters were calculated at each dosing regimen: *f*AUC, *f*C_max_, *f*AUC/MEM-KSP-1007 (M-K) MIC, *f*C_max_/M-K MIC, %*f*T>M K MIC, and %*f*T>C_T_ (see Tables S2 and S3 in the supplemental material, which show only *f*C_max_, *f*AUC, and %*f*T>MIC or C_T_ at representative regimens). The efficacy data of changes in log_10_ CFU/thigh were pooled for this analysis. The relationship between efficacy and each PK/PD parameter was evaluated by a sigmoid *E*_max_ model: *E* = *E*_0_ + (*E*_max_ × *D^N^*)/(ED_50_*^N^ + D^N^*), where *E*_0_ is the change in log_10_ CFU/thigh from baseline when *D* is 0, *E*_max_ is the maximum reduction in log_10_ CFU/thigh, *D* is each PK/PD parameter, ED_50_ is the *D* value required to achieve 50% of *E*_max_, and *N* is the Hill coefficient for the sigmoidicity of the dose-effective curve. %*f*T>C_T_ was investigated as the threshold concentration of 0.25, 0.5, 1, 2, and 4 μg/mL for CPE and 0.5, 1, 2, 4, and 8 μg/mL for CPAB. M-K MIC with fixed concentrations of 4, 8, and 16 μg/mL of KSP-1007 were described as M-K MIC at 4 μg/mL, M-K MIC at 8 μg/mL, and M-K MIC at 16 μg/mL, respectively. The magnitude of each PK/PD parameter associated with stasis or 1-log_10_ bacterial killing represents the value required to maintain the bacterial density at the same level or achieve a 1-log_10_ reduction from the bacterial burden at the start of treatment. In each regimen (q3h, q6h, q12h, and every 24 h [q24h]), ED_50_ (the effective dose corresponding to 50% of *E*_max_ − *E*_min_) values were calculated using a sigmoid *E*_max_ model fitted to the individual changes in log_10_ CFU/thigh (*n* = 4). The analysis described above was performed using Prism 8 or Prism 9 (GraphPad Software, LLC., Boston, MA).

### Statistical analysis

Dose responses were assessed by the Jonckheere-Terpstra test applied to individual changes in log_10_ CFU/thigh (*n* = 4) using the SAS program in the Stat Preclinica (Takumi Information Technology Inc., Tokyo, Japan). A *P* value <0.05 was considered to be significant.

## SUPPLEMENTAL MATERIAL

Supplemental material aac.01852-25-s0001.pdf Tables S1 to S4; Fig. S1 to S5; Supplemental methods.

ASM does not own the copyrights to Supplemental Material that may be linked to, or accessed through, an article. The authors have granted ASM a non-exclusive, world-wide license to publish the Supplemental Material files. Please contact the corresponding author directly for reuse.
