# Supplementary material for: Pharmacokinetics/pharmacodynamics of KSP-1007 in combination with meropenem against carbapenemase-producing gram-negative bacteria in a neutropenic murine thigh infection model
Source: Antimicrob Agents Chemother. 2026 Mar 3;70(4):e01852-25. doi: 10.1128/aac.01852-25 (PMC13041344; doi:10.1128/aac.01852-25)
Supplement: Supplemental material — Tables S1 to S4; Fig. S1 to S5; Supplemental methods. [file aac.01852-25-s0001.pdf]

Table S1. Efficacy data (change in $\log_{10}$ CFU/thigh) and ED_{50} values from an E_{max} model for each dosing interval in the thigh infection model

Strain		Changes in $\log_{10}$ CFU/thigh ± SD at 24 h after treatment initiation across dosing intervals			
Carbapenemase	Total daily doses	Dosing intervals			
Baseline at 2 h after infection		3 h	6 h	12 h	24 h
K. pneumoniae ATCC BAA-2344	Control (saline)	1.81 ± 0.39			
KPC-2	MEM 40 mg/kg/day	1.90 ± 0.25			
7.13 $\log_{10}$ CFU/thigh	+ KSP-1007 16 mg/kg/day	-0.40 ± 0.27	0.56 ± 0.37	-0.21 ± 1.01	0.45 ± 0.79
	+ KSP-1007 4 mg/kg/day	0.80 ± 0.12	1.27 ± 0.16	1.42 ± 0.15	1.50 ± 0.22
	+ KSP-1007 1 mg/kg/day	1.87 ± 0.34	1.75 ± 0.35	1.74 ± 0.46	1.75 ± 0.57
	ED_{50}	**7.65 mg/kg/day**	19.3 mg/kg/day	11.5 mg/kg/day	18.9 mg/kg/day
K. pneumoniae ATCC BAA-1902	Control (saline)	0.43 ± 1.06			
KPC-3	MEM 40 mg/kg/day	0.84 ± 0.91			
6.40 $\log_{10}$ CFU/thigh	+ KSP-1007 960 mg/kg/day	-1.21 ± 0.17	-1.18 ± 0.10	-1.14 ± 0.15	
	+ KSP-1007 240 mg/kg/day	-1.12 ± 0.18	-1.52 ± 0.31	-0.91 ± 0.33	-0.95 ± 0.59
	+ KSP-1007 60 mg/kg/day	-1.07 ± 0.13	-1.43 ± 0.33	-0.98 ± 0.30	-0.34 ± 0.69
	ED_{50}	12.1 mg/kg/day	**3.93 mg/kg/day**	18.1 mg/kg/day	50.6 mg/kg/day
K. pneumoniae CDC-113	Control (saline)	1.60 ± 0.19			
KPC-3	MEM 800 mg/kg/day	0.76 ± 0.32			
6.87 $\log_{10}$ CFU/thigh	+ KSP-1007 480 mg/kg/day	-1.97 ± 0.33	-1.67 ± 0.21	-1.43 ± 0.15	
	+ KSP-1007 240 mg/kg/day	-1.68 ± 0.20	-1.31 ± 0.14	-0.66 ± 0.29	
	+ KSP-1007 120 mg/kg/day	-1.41 ± 0.30	-1.05 ± 0.12	-0.41 ± 0.53	
	+ KSP-1007 60 mg/kg/day	-1.33 ± 0.11	-0.61 ± 0.14	-0.02 ± 0.33	
	ED_{50}	**22.7 mg/kg/day**	64.1 mg/kg/day	172 mg/kg/day	

Table S1−*Continued*

Strain<br>Carbapenemase<br>Baseline at 2 h after infection	Total daily doses	Changes in $\log_{10}$ CFU/thigh ± SD at 24 h after treatment initiation across dosing intervals			
		Dosing intervals			
		3 h	6 h	12 h	24 h
K. pneumoniae KUB3606	Control (saline)	2.60 ± 0.33			
KPC-38	MEM 800 mg/kg/day	1.98 ± 0.44			
6.96 $\log_{10}$ CFU/thigh	+ KSP-1007 960 mg/kg/day	-2.68 ± 0.19	-2.89 ± 0.38	-2.18 ± 1.06	
	+ KSP-1007 240 mg/kg/day	-2.57 ± 0.26	-1.86 ± 1.36	-1.17 ± 1.56	
	+ KSP-1007 60 mg/kg/day	-0.40 ± 1.66	0.18 ± 1.25	0.63 ± 1.35	
	ED_{50}	**57.6 mg/kg/day**	89.6 mg/kg/day	150 mg/kg/day	
K. pneumoniae KUB3166	Control (saline)	2.47 ± 0.28			
IMP-1	MEM 40 mg/kg/day	0.93 ± 0.35			
7.33 $\log_{10}$ CFU/thigh	+ KSP-1007 240 mg/kg/day	-0.57 ± 0.12	-0.35 ± 0.19	-0.20 ± 0.50	-0.01 ± 0.17
	+ KSP-1007 60 mg/kg/day	-0.24 ± 0.15	-0.06 ± 0.52	-0.48 ± 0.74	0.10 ± 0.59
	+ KSP-1007 15 mg/kg/day	0.20 ± 0.36	0.34 ± 0.48	0.26 ± 0.63	0.05 ± 0.56
	ED_{50}	**10.8 mg/kg/day**	24.5 mg/kg/day	13.1 mg/kg/day	32.5 mg/kg/day
K. pneumoniae ATCC BAA-2473	Control (saline)	2.29 ± 0.33			
NDM-1	MEM 40 mg/kg/day	0.56 ± 0.34			
6.94 $\log_{10}$ CFU/thigh	+ KSP-1007 960 mg/kg/day	-0.73 ± 0.08	-0.97 ± 0.18	-1.12 ± 0.09	
	+ KSP-1007 240 mg/kg/day	-1.05 ± 0.26	-0.76 ± 0.12	-0.10 ± 0.66	0.04 ± 0.90
	+ KSP-1007 60 mg/kg/day	-0.54 ± 0.27	-0.68 ± 0.63	0.47 ± 0.64	0.99 ± 0.05
	ED_{50}	52.7 mg/kg/day	**49.6 mg/kg/day**	244 mg/kg/day	255 mg/kg/day

Table S1−*Continued*

Strain<br>Carbapenemase<br>Baseline at 2 h after infection	Total daily doses	Changes in log_{10} CFU/thigh ± SD at 24 h after treatment initiation across dosing intervals			
		Dosing intervals			
		3 h	6 h	12 h	24 h
K. pneumoniae CDC-40	Control (saline)	2.07 ± 0.21			
VIM-27	MEM 400 mg/kg/day	1.04 ± 0.51			
7.32 log_{10} CFU/thigh	+ KSP-1007 480 mg/kg/day	-0.31 ± 0.20	-0.15 ± 0.43	0.21 ± 0.97	
	+ KSP-1007 240 mg/kg/day	-0.69 ± 0.58	-0.16 ± 0.61	0.39 ± 0.32	
	+ KSP-1007 120 mg/kg/day	-0.45 ± 1.81	0.56 ± 0.38	0.69 ± 0.10	
	+ KSP-1007 60 mg/kg/day	0.05 ± 0.61	0.60 ± 0.44	0.41 ± 0.75	
	ED_{50}	**30.0 mg/kg/day**	190 mg/kg/day	412 mg/kg/day	
K. pneumoniae CDC-68	Control (saline)	2.81 ± 0.30			
NDM-1, OXA-232	MEM 800 mg/kg/day	2.95 ± 0.18			
6.52 log_{10} CFU/thigh	+ KSP-1007 480 mg/kg/day	-1.69 ± 0.25	-0.89 ± 0.35	-0.14 ± 0.91	
	+ KSP-1007 120 mg/kg/day	-0.90 ± 0.52	0.31 ± 0.17	0.93 ± 0.47	
	+ KSP-1007 60 mg/kg/day	0.19 ± 0.54	1.03 ± 0.54	1.93 ± 0.26	
	+ KSP-1007 15 mg/kg/day	2.57 ± 0.23	2.06 ± 0.43	2.46 ± 0.14	
	ED_{50}	**51.9 mg/kg/day**	101 mg/kg/day	216 mg/kg/day	
K. pneumoniae CDC-138	Control (saline)	2.66 ± 0.08			
NDM-7	MEM 800 mg/kg/day	0.71 ± 0.68			
7.38 log_{10} CFU/thigh	+ KSP-1007 480 mg/kg/day	-1.61 ± 0.29	-1.53 ± 0.31	0.10 ± 0.91	
	+ KSP-1007 240 mg/kg/day	-1.32 ± 0.20	-0.56 ± 0.96	0.05 ± 0.63	
	+ KSP-1007 120 mg/kg/day	-1.10 ± 0.37	-0.28 ± 0.62	0.30 ± 0.75	
	+ KSP-1007 60 mg/kg/day	-0.13 ± 0.41	-0.43 ± 0.68	-0.20 ± 0.67	
	ED_{50}	**76.0 mg/kg/day**	108 mg/kg/day	14,500 mg/kg/day	

Table S1−*Continued*

Strain<br>Carbapenemase<br>Baseline at 2 h after infection	Total daily doses	Changes in $\log_{10}$ CFU/thigh ± SD at 24 h after treatment initiation across dosing intervals			
		Dosing intervals			
		3 h	6 h	12 h	24 h
A. baumannii CDC-277	Control (saline)	1.83 ± 0.26			
OXA-24, OXA-65	MEM 800 mg/kg/day	1.49 ± 0.24			
6.87 $\log_{10}$ CFU/thigh	+ KSP-1007 960 mg/kg/day	-3.11 ± 0.28	-2.45 ± 0.84	-2.67 ± 0.66	
	+ KSP-1007 240 mg/kg/day	-2.57 ± 0.63	-1.60 ± 1.63	-1.00 ± 1.33	0.39 ± 1.13
	+ KSP-1007 60 mg/kg/day	-0.81 ± 0.64	-0.10 ± 0.74	0.56 ± 1.18	0.36 ± 1.38
	ED_{50}	**66.3 mg/kg/day**	159 mg/kg/day	245 mg/kg/day	730 mg/kg/day
A. baumannii CDC-83	Control (saline)	0.69 ± 0.14			
NDM-1, OXA-23, OXA-69	MEM 800 mg/kg/day	0.91 ± 0.21			
7.61 $\log_{10}$ CFU/thigh	+ KSP-1007 960 mg/kg/day	-3.51 ± 0.99	-3.65 ± 0.36	-3.89 ± 0.91	
	+ KSP-1007 240 mg/kg/day	-2.90 ± 0.35	-2.08 ± 1.13	-0.68 ± 0.35	-2.31 ± 1.95
	+ KSP-1007 60 mg/kg/day	-0.86 ± 0.43	-0.45 ± 0.51	-0.10 ± 0.94	-0.45 ± 0.33
	ED_{50}	**106 mg/kg/day**	168 mg/kg/day	308 mg/kg/day	151 mg/kg/day
A. baumannii CDC-289	Control (saline)	1.94 ± 0.14			
OXA-66, OXA-72	MEM 800 mg/kg/day	2.04 ± 0.14			
7.14 $\log_{10}$ CFU/thigh	+ KSP-1007 480 mg/kg/day	-2.88 ± 0.38	-3.00 ± 0.23	-3.02 ± 0.89	-3.65 ± 0.38
	+ KSP-1007 240 mg/kg/day	-2.87 ± 0.83	-2.41 ± 1.48	-2.04 ± 1.90	-1.83 ± 2.23
	+ KSP-1007 60 mg/kg/day	-1.51 ± 0.89	-0.11 ± 0.09	0.71 ± 0.15	-0.50 ± 2.25
	ED_{50}	**39.0 mg/kg/day**	78.9 mg/kg/day	113 mg/kg/day	71.7 mg/kg/day

Table S1−*Continued*

Strain<br>Carbapenemase<br>Baseline at 2 h after infection	Total daily doses	Changes in $\log_{10}$ CFU/thigh ± SD at 24 h after treatment initiation across dosing intervals			
		Dosing intervals			
		3 h	6 h	12 h	24 h
A. baumannii CDC-301	Control (saline)	1.13 ± 0.30			
OXA-66, OXA-72	MEM 800 mg/kg/day	1.35 ± 0.22			
7.25 $\log_{10}$ CFU/thigh	+ KSP-1007 960 mg/kg/day	-2.56 ± 0.83	-2.17 ± 0.67	-1.60 ± 0.86	
	+ KSP-1007 240 mg/kg/day	-2.33 ± 0.93	-1.47 ± 1.74	-0.28 ± 0.32	0.21 ± 0.26
	+ KSP-1007 60 mg/kg/day	-0.97 ± 2.12	0.32 ± 0.30	0.60 ± 0.16	0.21 ± 0.34
	ED_{50}	**46.7 mg/kg/day**	158 mg/kg/day	406 mg/kg/day	536 mg/kg/day
A. baumannii CDC-293	Control (saline)	2.18 ± 0.14			
OXA-66, OXA-72	MEM 800 mg/kg/day	1.94 ± 0.18			
7.27 $\log_{10}$ CFU/thigh	+ KSP-1007 960 mg/kg/day	-3.27 ± 0.40	-3.12 ± 0.58	-1.24 ± 1.02	
	+ KSP-1007 240 mg/kg/day	-2.61 ± 0.54	-1.41 ± 0.92	0.26 ± 1.08	-2.02 ± 2.55
	+ KSP-1007 60 mg/kg/day	0.15 ± 0.32	0.38 ± 0.89	1.48 ± 0.14	1.70 ± 0.14
	ED_{50}	**92.4 mg/kg/day**	149 mg/kg/day	607 mg/kg/day	167 mg/kg/day

Table S2. Pharmacokinetic parameters (fC_{max} and fAUC) and PK/PD parameters (%fT>C_T) of KSP-1007 across dosing regimens

Dose (mg/kg)	Interval (h)	fC_{max} (μg/mL)	fAUC (μg·h/mL)	%fT>C_T					
				0.25 μg/mL	0.5 μg/mL	1 μg/mL	2 μg/mL	4 μg/mL	8 μg/mL
480	12	312	449	58.4	48.2	38.4	30.0	23.7	19.0
240	6	156	449	97.2	77.6	60.5	47.7	38.2	30.5
120	3	78.8	448	100	100	98.8	78.2	62.0	47.9
240	24	156	112	24.1	19.2	15.0	11.9	9.52	7.60
120	12	77.9	112	38.5	30.0	23.7	19.0	15.2	11.8
60	6	39.0	112	60.5	47.7	38.2	30.5	23.6	17.2
30	3	19.7	112	98.9	78.3	61.9	47.8	34.8	22.2
60	24	39.0	28.1	15.0	11.9	9.51	7.59	5.90	4.30
30	12	19.5	28.1	23.7	19.0	15.2	11.8	8.60	5.48
15	6	9.75	28.0	38.2	30.5	23.6	17.2	11.0	4.18
7.5	3	4.92	28.0	62.0	47.9	34.8	22.2	8.61	0.00

Table S3. Pharmacokinetic parameters (fC_{max} and fAUC) and PK/PD parameters (%fT>MIC) of MEM across dosing regimens

Dose (mg/kg)	Interval (h)	fC_{max} (μg/mL)	fAUC (μg·h/mL)	%fT>MIC[a]														
				0.015	0.03	0.06	0.12	0.25	0.5	1	2	4	8	16	32	64	128	256
100	3	85.4	424	100	100	100	95.9	86.2	77.1	68.0	58.9	49.7	40.5	31.2	21.5	10.4	0.00	0.00
50	3	42.7	212	100	100	95.8	86.8	77.1	68.0	58.9	49.7	40.5	31.2	21.5	10.3	0.00	0.00	0.00
5	3	4.27	21.2	83.8	74.7	65.6	56.5	46.8	37.5	28.2	18.3	4.76	0.00	0.00	0.00	0.00	0.00	0.00

[a]The numbers shown below indicate the corresponding MIC values in μg/mL.

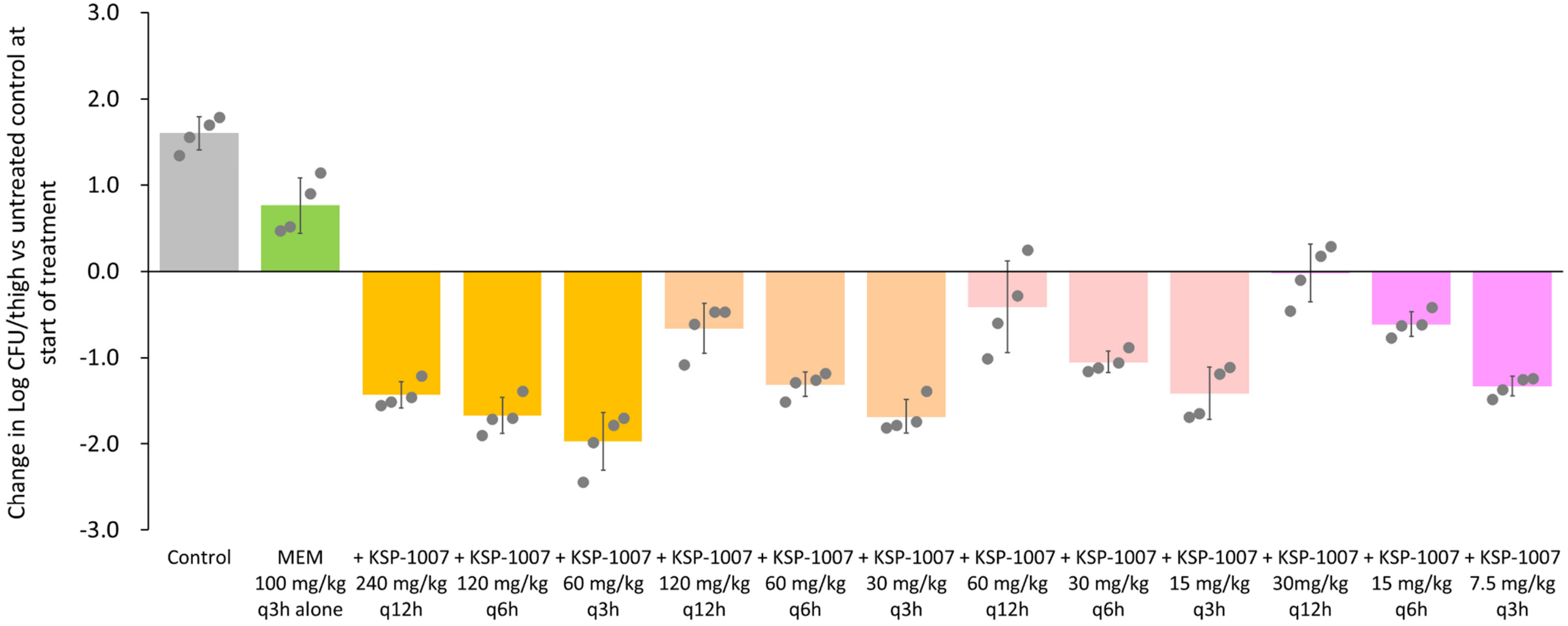

Figure S1. Activity of MEM at 100 mg/kg q3h alone and in combination with KSP-1007 against *K. pneumoniae* CDC-113 (KPC-3 producer) in a murine thigh infection model. Individual (circle) and mean (bar) bacterial growth or reduction in $\log_{10}$ CFU/thigh ± the standard deviation at 24 h from that at treatment initiation. The mean bacterial burden in the thighs at the start of treatment was 6.87 $\log_{10}$ CFU/thigh. Treatments were started 2 h after infection and continued for 24 h by the subcutaneous route ($n = 4$).

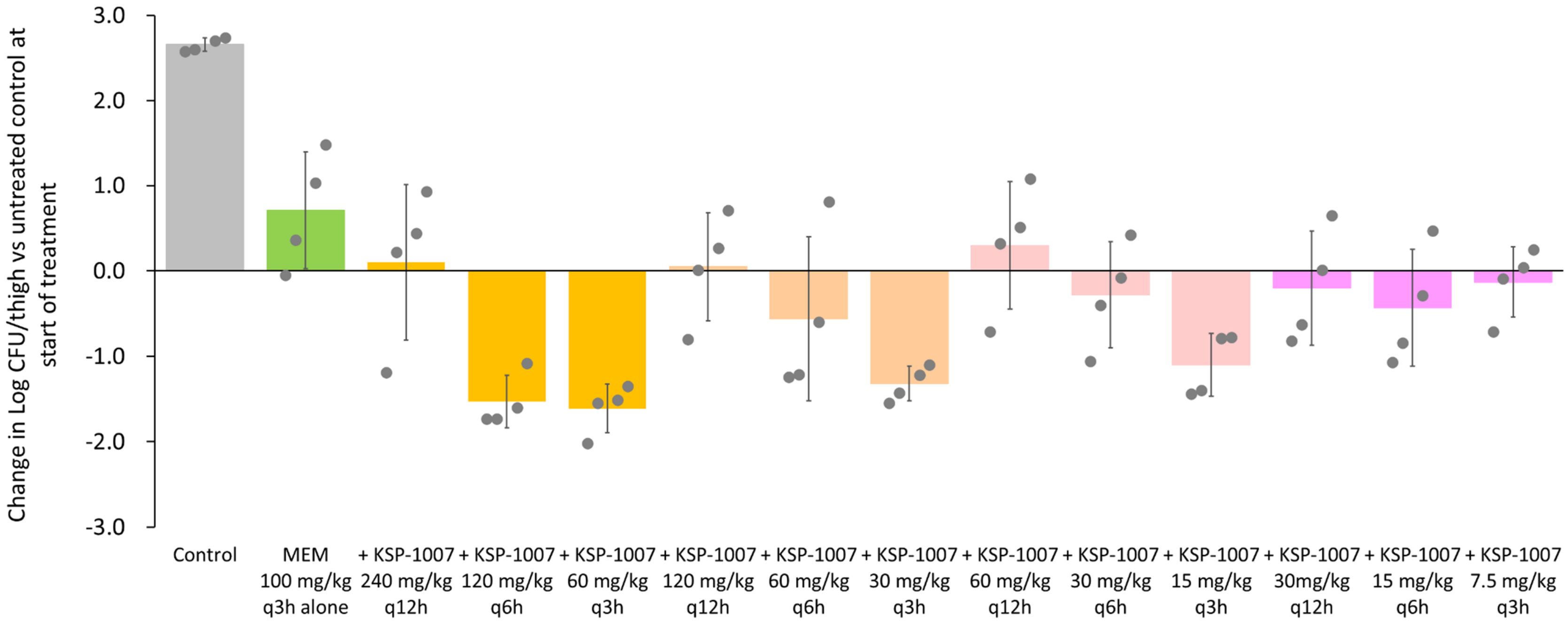

Figure S2. Activity of MEM at 100 mg/kg q3h alone and in combination with KSP-1007 against *K. pneumoniae* CDC-138 (NDM-7 producer) in a murine thigh infection model. Individual (circle) and mean (bar) bacterial growth or reduction in log_{10} CFU/thigh ± the standard deviation at 24 h from that at treatment initiation. The mean bacterial burden in the thighs at the start of treatment was 7.38 log_{10} CFU/thigh. Treatments were started 2 h after infection and continued for 24 h by the subcutaneous route (*n* = 4).

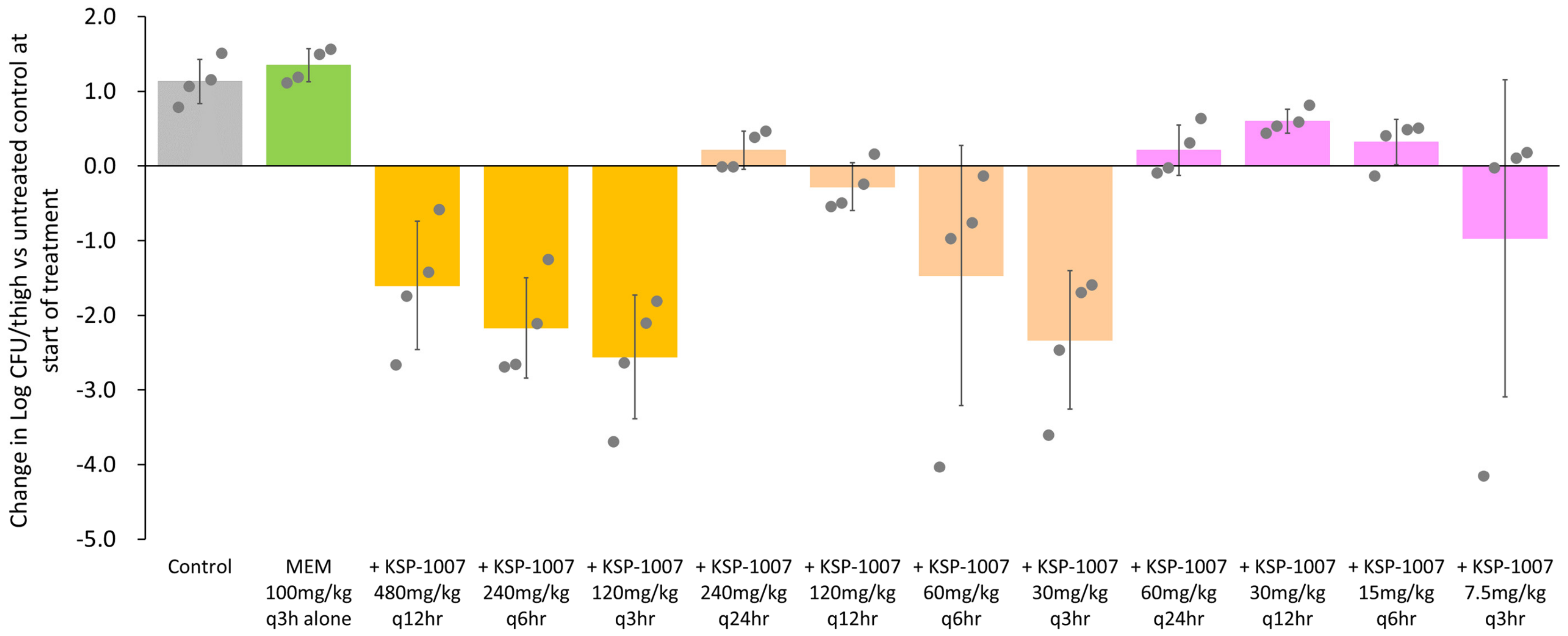

Figure S3. Activity of meropenem at 100 mg/kg q3h alone and in combination with KSP-1007 against *A. baumannii* CDC-301 (OXA-66 and OXA-72 producer) in a murine thigh infection model. Individual (circle) and mean (bar) bacterial growth or reduction in $\log_{10}$ CFU/thigh ± the standard deviation at 24 h from that at treatment initiation. The mean bacterial burden in the thighs at the start of treatment was 7.25 $\log_{10}$ CFU/thigh. Treatments were started 2 h after infection and continued for 24 h by the subcutaneous route ($n = 4$).

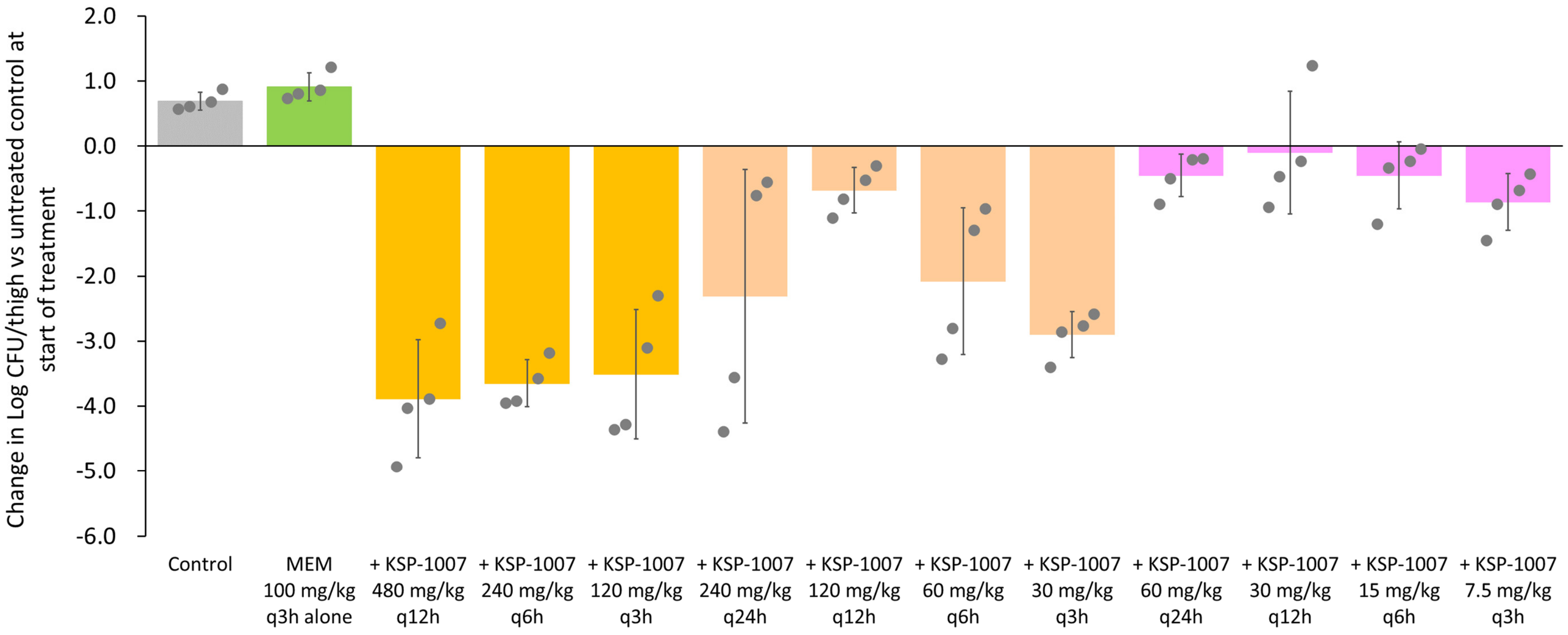

Figure S4. Activity of meropenem at 100 mg/kg q3h alone and in combination with KSP-1007 against *A. baumannii* CDC-83 (NDM-1, OXA-23, and OXA-69 producer) in a murine thigh infection model. Individual (circle) and mean (bar) bacterial growth or reduction in $\log_{10}$ CFU/thigh ± the standard deviation at 24 h from that at treatment initiation. The mean bacterial burden in the thighs at the start of treatment was 7.61 $\log_{10}$ CFU/thigh. Treatments were started 2 h after infection and continued for 24 h by the subcutaneous route ($n = 4$).

Table S4. Efficacy of MEM/KSP-1007 in the *A. baumannii* CDC-277 lung infection model

Dosage regimen (n = 8)	KSP-1007 Dosage regimen	Log_{10} CFU/lung		Change in log_{10} CFU/lung[b]
		Mean	SEM[a]	
Control	–	8.17	0.11	2.96
Colistin 30 mg/kg q12h	–	4.73	0.35	-0.48
MEM 100 mg/kg q3h	–	5.32	0.21	0.11
MEM 100 mg/kg q3h plus KSP-1007 240 mg/kg/day	30 mg/kg q3h	1.35[c]	0.06	-3.86
	60 mg/kg q6h	1.25[c]	0.11	-3.96
	120 mg/kg q12h	1.04[c]	0.04	-4.17
MEM 100 mg/kg q3h plus KSP-1007 480 mg/kg/day	60 mg/kg q3h	1.04[c]	0.04	-4.17
	120 mg/kg q6h	1.13[c]	0.07	-4.08
	240 mg/kg q12h	1.00[c]	0.00	-4.21

[a]SEM, standard error of the mean.
[b]The log_{10} difference in bacterial counts (CFU/lung) from that at treatment initiation (5.21 log_{10} CFU/lung).
[c]Significantly different from MEM 100 mg/kg q3h (P <0.05 by a one-way ANOVA and Dunnett's test using GraphPad Prism Software).

(A) (B)

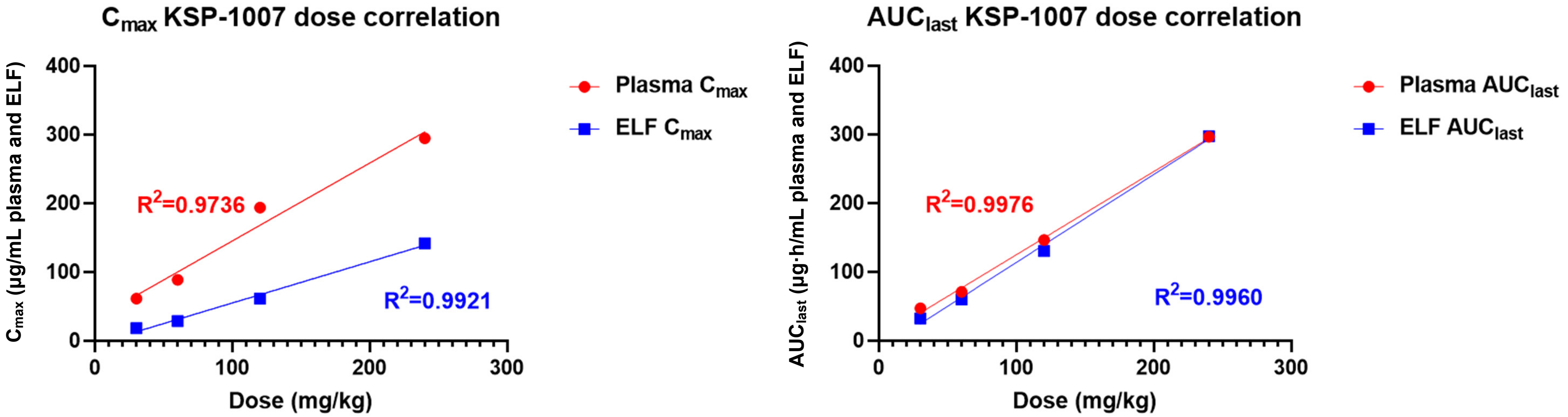

Figure S5. Dose correlation of KSP-1007 at subcutaneous administration with C_{max} (A) or AUC_{last} (B) in plasma and ELF of the murine *A. baumannii* CDC-277 lung infection model.

Supplemental Materials and Methods in the murine lung infection model

Materials

KSP-1007 was synthesized at Sumitomo Pharma Co., Ltd.

Meropenem (Meropenem powder for Intravenous Injection, Savior Lifetec Corporation), Colistin Sulfate (C4461, Sigma, USA), Cilastatin (BLD Pharmatech Ltd., Shanghai, China), and Cyclophosphamide monohydrate (C0768, Sigma, USA).

Murine lung infection model

All animal experiments involving the lung infection model were conducted by Pharmacology Discovery Services (PDS), and the procedures were approved by the PDS Institutional Animal Care and Use Committee.

Seven- to eight-week-old female BALB/c mice (BioLASCO, Taiwan) were rendered immunocompromised by intraperitoneal administration of cyclophosphamide at 150 mg/kg and 100 mg/kg on days 4 and 1 before infection, respectively. The organism was cultured in 20 mL Brain Heart Infusion (BHI) at 35–37°C with shaking (250 rpm) for 6 h, followed by subculturing 1 mL in 99 mL fresh BHI and incubating under the same conditions for 16 h. Bacterial cells were pelleted by centrifugation (3,500 × *g*) for 15 min and resuspended in phosphate-buffered saline (PBS). Optical density was measured at 620_{nm} and used to adjust the suspension to the target bacterial concentration. Cyclophosphamide-treated neutropenic mice were intranasally inoculated with the bacterial suspension (approximately 1×10^6 CFU/mouse, 10 µL per nostril) under anesthesia 2 h before therapy. Mice were then subcutaneously treated with MEM at 100 mg/kg q3h for 24 h, either alone or in combination with KSP-1007 administered at 3-, 6-, or 12-h dosing intervals (240 and 480 mg/kg/day). Cilastatin (100 mg/kg) was co-administered with MEM at each dosing. Colistin at 30 mg/kg was subcutaneously administered q12h for 24 h, similar to previously reported methods (1). The control group was subcutaneously administered sterile saline q3h for 24 h. Mice were sacrificed 2 and 26 h after infection with CO_2 asphyxiation, and the lungs were aseptically harvested and homogenized in 1 mL of sterile PBS using a Polytron homogenizer. Bacterial burden was assessed using serial dilutions and plating on MacConkey II agar, following the same procedure as that described for the thigh infection model.

Measurement of KSP-1007 concentrations in plasma and lung ELF.

A single dose of KSP-1007 was administered subcutaneously 2 h after infection: 30, 60, 120, or 240 mg/kg in the lung infection model. Blood samples were collected at eight time points post-dosing via cardiac puncture under anesthesia and placed in a heparinized tube (0.083, 0.25, 0.5, 1, 2, 4, 8, and 24 h). Plasma was separated by centrifugation and stored at −70°C until analyzed. Bronchoalveolar lavage fluid (BALF) was collected at the same time points as blood sampling by instilling 0.5 mL PBS through a tracheal cannula, followed by the recovery of approximately 0.2–0.3 mL BALF. BALF samples were centrifuged, and the supernatant was stored at −70°C until analyzed. The concentrations of KSP-1007 in both plasma and BALF were measured by liquid chromatography-tandem mass spectrometry. The concentration in ELF was corrected by measuring urea concentrations in BALF and plasma (2).

The pharmacokinetic parameters of KSP-1007 were estimated using a non-compartmental analysis. Analyses were performed using Phoenix WinNonlin version 8.3 (Certara L.P., Radnor, PA).

Supplemental References

1. Lim AL, Miller BW, Lin Z, Fisher MA, Barrows LR, Haygood MG, Schmidt EW. 2023. Resistance mechanisms for Gram-negative bacteria-specific lipopeptides, turnercyclamycins, differ from that of colistin. Microbiol Spectr 11:e02306-23.
2. Rennard SI, Basset G, Lecossier D, O'Donnell KM, Pinkston P, Martin PG, Crystal RG. 1986. Estimation of volume of epithelial lining fluid recovered by lavage using urea as marker of dilution. J Appl Physiol 60:532-538.
